# Effect of *APOL1* disease risk variants on *APOL1* gene product

**DOI:** 10.1042/BSR20160531

**Published:** 2017-04-28

**Authors:** Shabirul Haque, Gauri Patil, Abheepsa Mishra, Xiqian Lan, Waldemar Popik, Ashwani Malhotra, Karl Skorecki, Pravin C. Singhal

**Affiliations:** 1 Renal Molecular Research Laboratory, Feinstein Institute for Medical Research, Hofstra North Shore LIJ Medical School, New York, U.S.A.; 2 Center for Health Disparity Reseaerch, Meharry Medical College, Nashville, TN, U.S.A.; 3 Nephrology and Molecular Medicine, Technion, Institute of Technology and Rambam Medical Center, Haifa, Israel

**Keywords:** APOL1, APOL1 gene product, APOL1 disease risk variants

## Abstract

Gene sequence mutations may alter mRNA transcription, transcript stability, protein translation, protein stability and protein folding. Apolipoprotein L1 (APOL1) has two sets of sequence variants that are risk factors for kidney disease development, APOL1G1 (substitution mutation) and APOL1G2 (deletion mutation). Our present study focuses on the impact of these variants on *APOL1* mRNA transcription and translation. APOL1 plasmids (EV, G0, G1 and G2) were transfected into human embryonic kidney (HEK) 293T cells. APOL1 variant expression was observed to be significantly lower than that of APOL1G0. Podocyte cell lines stably expressing APOL1 transgenes also showed lower levels of APOL1 expression of APOL1 variants (G1 and G2) compared with APOL1G0 by Western blotting and FACS analysis. The enhanced expression of GRP78 by podocytes expressing APOL1 variants would indicate endoplasmic reticulum (ER) stress. Bioinformatics evaluation using two different programs (MUPro and I-Mutant 2.0) predicted that APOL1 variants were less stable than APOL1G0. Concomitant with protein levels, *APOL1* mRNA levels were also depressed following induction of APOL1 variant compared with APOL1G0 in both proliferating and differentiated podocytes. *APOL1* mRNA transcript stability was tested after actinomycin D pulsing; *APOL1G1* and *APOL1G2* mRNAs transcript decayed 10–15% and 15–20% (within a period of 0.5–3 h) respectively. Our data suggest that down-regulated APOL1 protein expression in APOL1 variants is due to compromised transcription and decay of the APOL1 variant transcripts.

## Introduction

Apolipoprotein L1 (APOL1) is a protein encoded by *APOL1* gene [1]. It has two variants APOL1G1 and APOL1G2 (Jeffrey B. Kopp et al. 2011). Both variants increase the risk for chronic kidney disease (CKD) and end-stage renal disease (ESRD) in patients of Sub-Saharan African descent [2,3]. The APOL1G1 variant comprises two substitution mutations at amino acid position 342 (S→G) and at amino acid position 384 (I→M), while the APOL1G2 variant comprises two amino acids deletion (388 N 389 Y). DNA mutation within any gene can lead to altered transcription and translation, and to some extent, could also modify the regulation of other genes as well. The mutation may impact protein levels and activity with an incomplete or complete ‘gain of function’ or ‘loss of function’. It is important to establish the relative contribution of altered transcription, translation and protein instability to any change in activity caused by mutations. For example, the deletion of a single amino acid (Phe^508^) from PGP-3 results in the misfolding and degradation of the cystic fibrosis transmembrane conductance regulator (CFTR) Cl–channel, where PGP-3^∆Phe508^ was post-transcriptionally destabilized, resulting in the lowering of protein expression within the total and apical membrane compared with the wild-type protein [4]. GFP is a stable protein and mutation at amino acids 422–461 results in the destabilization and a decrease in half-life of protein [5]. Human *dopamine receptor D_2_* (DRD_2_) gene mutations lead to decrease in mRNA stability and protein translation [6]. Mutations in the human polyglutamine androgen receptor protein result in a reduced life span of the protein compared with wild-type androgen receptor; the shorter protein life span has been attributed to degradation of the protein by the ubiquitin-proteasome machinery [7]. Several temperature-sensitive mutants of GFP have been developed and protein stabilities were evaluated at different temperatures in soluble and insoluble fractions of cell lysates. These studies yielded mutant gene products with almost 70% loss/degradation at 37°C, while the same mutant was completely stable at 30°C in cell culture settings. These findings demonstrate that cell culture temperature has a great impact on protein stability regulation [8]. Bioinformatics approaches to predict and determine the protein structure and function in mutants [9] indicate that point-deletion mutants predicted a loss in enzymatic activity, with a marked alteration in protein activity. A recent publication showed that hepatocytes compromised mRNA expression of APOL1 variants compared with APOL1G0 [10]. However, the involved mechanism of down-regulation of APOL1 expression was not investigated in these studies.

Our current study examined the effect of APOL1 variant (G1/G2) mutations on the regulation of *APOL1* mRNA transcription and APOL1 protein translation and stability. We report that APOL1 variant (G1/G2) mutations caused altered *APOL1* mRNA transcription and transcript stability. Protein expression of APOL1 variants was also dysregulated compared with APOL1G0 and this dysregulation was accompanied by indices consistent with destabilization of APOL1 protein.

## Materials and methods

### Cell culture

Immortalized human podocytes proliferate at a permissive temperature (33°C) and enter growth arrest after transfer to the non-permissive temperature (37°C). At 37°C, podocytes differentiate into mature podocytes. Growth medium contained RPMI 1640 supplemented with 10% FBS, 1× Pen-Strep, 1 mM L-glutamine and 1× ITS (Thermo Fisher Scientific) to promote expression of T antigen. ITS-free culture medium was used for differentiation of podocytes. Human embryonic kidney (HEK cell line, 293T) cells were cultured at 37°C in DMEM supplemented with 10% FBS, 1× Pen-Strep and 1 mM L-glutamine.

### Overexpression of APOL1 protein in 293T cells

HEK 293T cells were expanded and plated on 100-mm plate. Cells were transfected with an equal amount of APOL1 empty vector, APOL1G0, APOL1G1 and APOL1G2. In brief, an equal number of cells were plated and incubated overnight at 37°C. Equal copy number/amount of plasmid was transfected using Effectene Transfection Regent Kit (Qiagen cat # 301425). Next day, cells were washed and harvested for cell lysate extraction.

### Protein expression by Western blotting

Cultured cells were washed with cold PBS and harvested for cell lysates in RIPA buffer (1× PBS, pH 7.4, 0.1% SDS, 1% NP-40, 0.5% sodium deoxycholate, 1.0 mM sodium orthovanadate, 10 μl of protease inhibitor cocktail (100×, Calbiochem)/1 ml of buffer and 100 μg/ml PMSF). Protein concentrations of the cell lysate were estimated by Bio–Rad assay. Protein (20–30 μg) was loaded and electrophoresed on 10–12% SDS/PAGE. Electrophoresed proteins were transferred to a PVDF membrane (Bio–Rad, Hercules, CA) overnight in the cold room. Transferred membrane blots were blocked with PBS-Tween (0.1%) with 5% non-fat milk for 2 h. Primary antibodies (1:1000 dilution) were added for overnight incubation at 4°C. Blots were washed with PBS-Tween 3× for 5 min. Then horseradish peroxidase–conjugated secondary antibodies (1:2000, Santa Cruz Biotechnology), were added and further incubated for 1 h at room temperature. Then, the blots were developed using ECL solution (Pierce) and exposed to X-ray film (Eastman Kodak Co., Rochester, NY). Developed blots were scanned using the Bio–Rad imaging system and quantification of band intensity was analysed by ImageJ. Of note, APOL1 protein expression was also confirmed by using APOL1 antibodies from two different suppliers, anti-lipoproteinL1 (apoL1) (297-309) in goat from Acris GmbH, Germany and apoL1 [(A-3) sc-390440] mouse monoclonal IgG_1_ from Santa Cruz Biotechnology.

### RNA extraction and cDNA preparation

Total RNA was extracted from cultured human podocytes using TRIzol reagent (Thermo Fisher Scientific) method. RNA concentration was estimated by Epoch Biotek spectrophotometer. RNA (4–5 μg) was used for cDNA synthesis using first-strand synthesis system (Thermo Fisher Scientific) standard protocol. Oligo (dT) primer was used for cDNA synthesis. For testing mRNA stability, cell lines were pulsed with actinomycin D (Sigma–Aldrich) and then total RNA was extracted at different time points.

### Detection of proteasomal activity

APOL1 cell lines (Vector, G0, G1 and G2 variants) were lysed in buffer (20 mM Tris/HCl (pH 7.2), 50 mM NaCl, 2 mM MgCl_2_, 0.1% Nonidet P-40) and protease inhibitor cocktail complete (Roche Molecular Biochemicals, Germany). Protein estimation was carried out by the Bio–Rad assay. Approximately 200–300 µg protein from each cellular lysate was incubated in quadruplet using substrate buffer (20 mM Tris/HCl (pH 7.2), 25 mM KCl, 10 mM NaCl, 1 mM MgCl_2_ and 0.1 mM EDTA) containing 100 µM synthetic fluorogenic peptide substrate (Suc-LLVYAMC, Enzo cat # BMP-P802) for 60 min at 37°C in dark. Fluorescence was determined with a plate reader (SLT-Lab Instruments, Crailsheim, Germany) using an excitation wavelength of 390 nm and the emission spectrum at 460 nm.

### Real-time q-PCR by Taq-Man

Primers sequences, probe number, gene accession number from universal probe library (UPL) are described in Table 1. Each sample was analysed in duplicates/triplicates using cDNA as a template. Eurogentec master (2×) mixes were used for PCR mixture. Data were analysed on RQ manager version (1.2.1). Fold changes were calculated by vector over APOL1G0, APOL1G1 and APOL1G2 values. The data are expressed as relative mRNA expression with reference to control sample, normalized to an endogeneous reference gene, *GAPDH*.

**Table 1:** Human primers from UPL (Roche)

Genes	Accession #	Probe #	Sequences
*APOL1*	AF305428.1	66	For: 5′-TGATAATGAGGCCTGGAACG-3′
			Rev: 5′-GGTTGTCCAGAGCTTTACGG-3′
*GAPDH*	NM_002046.3	60	For: 5′-AGCCACATCGCTCAGACAC-3′
			Rev: 5′-GCCCAATACGACCAAATCC-3′
*GRP78*	AF216292.1	6	For: 5′-GATAAAGAAAAGCTGGGAGGTAAA-3′
			Rev: 5′-CAGCTTTTTCCATGGTCTCC-3′
*XBP1*	NM_005080.3	37	For: 5′-GGAGTTAAGACAGCGCTTGG-3′
			Rev: 5′-CACTGGCCTCACTTCATTCC-3′
*ATF6*	AB015856.1	19	For: 5′-TTGGGACATCAACAACCAAA-3′
			Rev: 5′-GTCCACTGACCGAGGAGATG-3′
*GADD153*	S40706.1	11	For: 5′-GCCTTTCTCTTCGGACACTG-3′
			Rev: 5′-TCTTGCAGGTCCTCATACCA-3′

Transcript (mRNA) expression of active misfolding markers (GRP78, XBP1, ATF6 and GADD153) in vector and APOL1 variants (G0, G1 and G2) was also conducted.

### Cellular staining and FACS analysis

Cultured stable APOL1 podocytes were washed with 1× PBS then fixed for 15 min in 3% PFA at room temperature. Cells were washed with 3× PBS, permeabilized by adding ice-cold 90% methanol for 2 h at –20°C. Cells were washed an additional three times by centrifugation and then incubated with anti-APOL1 antibodies (Acris GmbH, Germany) at 1:100 dilution for 2 h. Cells were washed three times and Alexa Flour 488 secondary antibodies added at 1:200 dilution for 1 h at room temperature in the dark. After three additional washes, cells were fixed with PFA and analysed by flow cytometry (BD LSRFortessa). Data were analysed by FlowJo.

### Bioinformatics programs

#### MUPro

MUpro is a set of machine learning programs to predict how single-site amino acid mutation affects protein stability [11]. This program calculates using two machine learning approaches: support vector machines and neural networks. Training was conducted for both approaches on a large mutation dataset and yielded accuracies above 84% via 20-fold cross-validation. An advantage of these machine learning approaches for the current study is that they do not require tertiary structures to predict protein stability changes. Experimental results show that the prediction accuracy using sequence information alone is comparable with those using tertiary structures. Despite the unavailability of protein tertiary structures, accurate predictions can be inferred. However, if we are able to provide tertiary structures, this method would yield improved predictions. Prediction of the sign of energy changes using support vector machines and neural networks and effects of mutation on protein stability and a confidence score between –1 and 1 is used to determine the confidence of the prediction. Score less than 0 means that mutation is expected to decrease the protein stability. Lower the score, more confident the prediction is. A score greater than 0 means that the mutation is expected to increase the protein stability. Larger the score, more confident the prediction is. The accuracy for support vector machine is 84.2%, while the accuracy for support vector machine using tertiary structure information is 84.5%.

#### I-Mutant 2.0

I-Mutant 2.0 is a support vector machine based web server for the prediction of protein stability changes resulting from single-site mutations. The tool was trained on a dataset derived from ProTherm [12–15] that is presently the most comprehensive database of experimental data on protein mutations. This machine learning approach can evaluate the stability changes of single-site mutations starting from the resolved tertiary protein structure or from protein sequence. I-Mutant2.0 correctly predicts whether the protein mutation stabilizes or destabilizes the protein in 80% of the cases when the 3D structure is known and 77% of the cases when only protein sequence is available. The **DDG** value is calculated from the unfolding Gibb’s free energy value of the mutated protein minus the unfolding Gibb’s free energy value of the wild-type (kcal/mol).

### Statistical analysis

To compare the mean values between two groups, the unpaired *t* test was used. Statistical significance is defined as *P*<0.05. All results are shown as mean ± S.D. The number of data points for each experiment is given in figure legends. At least three different sets of experiments were carried out for each experimental condition. *P*-values are indicated as *P<0.05, **P<0.01, ***P<0.001.

## Results

### APOL1 expression in HEK 293T cells

**Figure 1:**
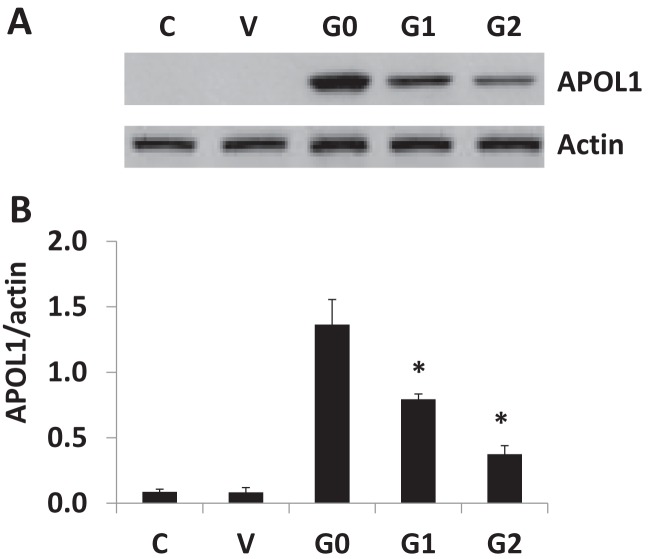
Overexpression of APOL1 into 293T cells by plasmid An equal amount of APOL1 plasmids (vector, APOL1 G0, APOL1 G1 and APOL1 G2) were transfected into 293T cells using Effectene Transfection Regent Kit. After 24 h, cells were harvested and cell lysates were prepared in RIPA buffer. An equal amount of protein was subjected to electrophoresis and transferred on to the PVDF membrane. Blots were probed with APOL1 antibody for detection of APOL1 protein expression and blots were stripped and re-probed for actin protein as a loading control (**A**). Densitometric scanning of blots is displayed as a bar graph (**B**). Results represent (mean ± S.D.); *P<0.05 compared with G0.

Mutations (either substitution or deletion) may modify protein characteristics on several levels. Our focus here is to understand whether APOL1 disease risk variant mutations impact the stability of APOL1 variant protein compared with wild-type APOL1 (G0). To address this important question, plasmids of a vector, APOL1G0, APOL1G1 and APOL1G2 were transfected into HEK 293T cells and incubated for 24 h at 37°C. APOL1 protein expression was analysed by Western blotting and densitometric analysis (Figure 1A,B). Our data indicate that the substitution mutations lead to lower expression of APOL1G1 compared with APOL1G0. The APOL1G2 further down-regulated APOL1 expression when compared with APOL1G1. Taken together, APOL1 expressions manifest phenotypically as APOL1G0 > APOL1G1 > APOL1G2.

### APOL1 expression in stable APOL1 podocyte cell lines during proliferative stage

**Figure 2:**
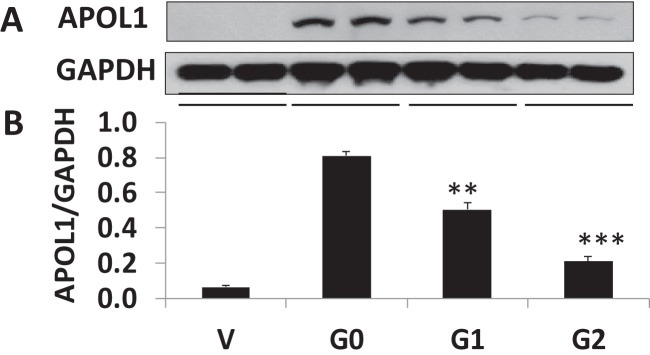
APOL1 protein expression in human APOL1 stable podocyte cell lines within proliferating cultured cells APOL1 cells were cultured and expanded at 33°C. Proliferating podocyte cells were harvested and cell lysates were prepared in RIPA buffer. Equal amount of proteins were subjected to electrophoresis and transferred on to membrane (*n*=3). Blots were probed for APOL1 protein (antibody source: Acris GmbH) and blots were stripped and re-probed for GAPDH (**A**). Densitometric scans of blots were quantified by ImageJ and plotted as a bar graph (**B**). *P<0.05 compared with G0, **P<0.01 compared with G0, ***P<0.001 compared with G0.

Immortalized human podocytes proliferate at 33°C and differentiate at 37°C. It is well established that mutations impair protein stability. Incubation temperature impacts protein stability. Therefore, podocyte cell lines APOL1G0, APOL1G1 and APOL1G2 were expanded at 33°C. From each condition, cell lysates were prepared and Western blotting performed using APOL1 antibody from Acris GmbH (Figure 2A). Densitometry analysis is presented in Figure 2B. These findings were further confirmed by using APOL1 antibody from Proteintech (representative blots, Supplementary Figure S1A) and calculated values of densitometric measurements are shown as bar graphs (Supplementary Figure S1B). We used another supplier of the APOL1 antibody as well from Santa Cruz (representative blots are shown in Supplementary Figure S1C) and pooled densitometric scan values are displayed as bar graphs (Supplementary Figure S1D). Even at 33°C, APOL1G1 expression was lower than APOL1G0. APOL1G2 expression was even lower than APOL1G1. Summarized APOL1 expression in podocytes at the proliferative temperature of 33°C showed APOL1G0 > APOL1G1 > APOL1G2. All three sources (suppliers) for antibodies showed similar protein expressions of APOL1 expression in APOL1G0 and APOL1 variants.

### APOL1 expression in stable APOL1 podocytes under differentiation

**Figure 3:**
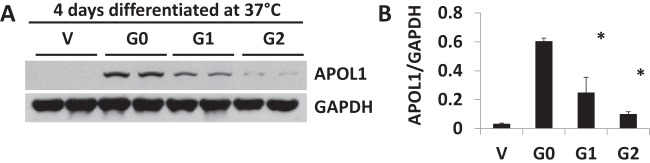
APOL1 protein level in APOL1 stable podocyte cell lines in partially differentiated cells Stable APOL1 cells were cultured and expanded at 33°C. Cells were differentiated at 37°C for 4 days and the partially differentiated podocytes were harvested. Cell lysates were prepared in RIPA buffer (*n*=3). Equal amounts of proteins were loaded for electrophoresis and transferred on to a membrane. Blots were probed for APOL1 protein and blots were stripped and re-probed for GAPDH. Representative Western blots (**A**) and bar graph of pooled densitometric data (**B**) are shown. Data are expressed as mean ± S.D. *P<0.05 compared with G0.

Podocytes differentiate at 37°C within 10–12 days. During this process of podocyte differentiation, several podocyte molecular markers are expressed. As we showed above, APOL1G1 and APOL1G2 podocytes have lower APOL1 expression compared twith APOL1G0. To delineate whether the differences in gene product expression between APOL1 G1 and G2 compared with G0 are consistent with the process of podocyte differentiation, we harvested cultured podocytes at days 4, 8 and 12. Cell lysates were prepared from each time point and conditions. Calculated densitometric scanned data are displayed as bar graphs. Data at 4 days are shown in Figure 3A,B. Representative gels and bar graph data at 8 and 12 days are shown in Supplementary Figure S2A–D. These data indicate that both APOL1G1 and APOL1G2 have lower expression of APOL1 when compared with APOL1G0 throughout the differentiation process. Podocyte differentiation has no impact on these differences in APOL1 expression and stabilization. Thus, APOL1 expression manifests as APOL1G0 > APOL1G1 > APOL1G2 in differentiated podocytes similar to proliferated podocytes.

### APOL1 expression by FACS in stable APOL1 podocyte cell lines

**Figure 4:**
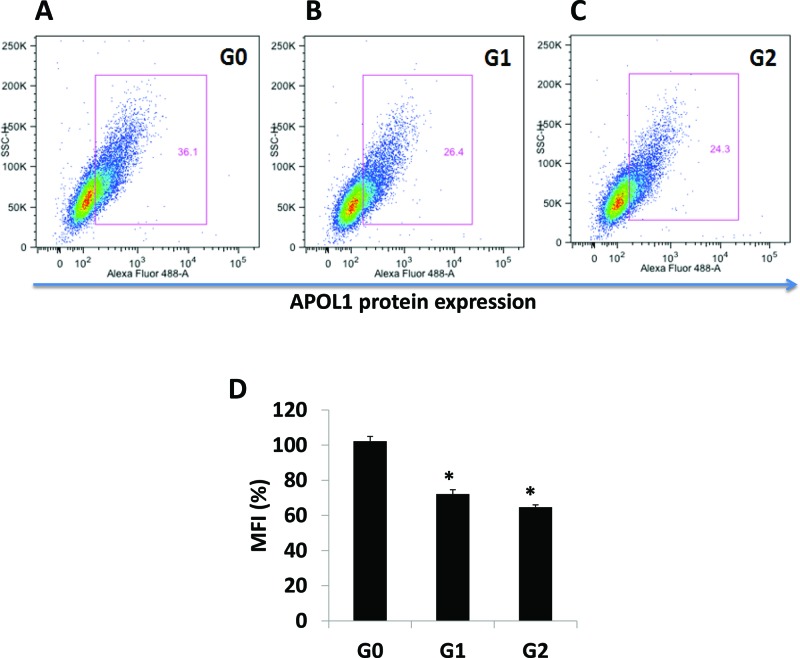
APOL1 expressions in proliferating APOL1 podocytes Stable podocytes were expanded at 33°C. Cells were harvested by trypsinization. Cells were stained with APOL1 antibodies. Stained cells were subjected to screening at flow cytometer (BD LSRFortessa). FACS data were analysed by FlowJo. Data are expressed as a bar graph (mean ± S.D.). *P<0.05 compared with G0.

All stained cells were subjected to run through FACS analysis. Data were analysed by a program FlowJo as illustrated in APOL1G0 (Figure 4A), APOL1G1 (Figure 4B) and APOL1G2 (Figure 4C). Cumulative data are represented as a bar graph (Figure 4D). These findings indicate that both APOL1G1 and APOL1G2 display attenuated expression when compared with APOL1G0. Summarized APOL1 expression appears as APOL1G0 > APOL1G1 > APOL1G2. These data complement the Western blotting results.

### Role of proteasomal activity in APOL1 expression

To determine whether alterations in proteasomal activity contributing to the modification of APOL1 expression, cellular lysates of a vector, APOL1G0, APOL1G1 and APOL1G2 were assayed for proteasomal activity (*n*=4). APOL1G0, APOL1G1 and APOL1G2 displayed attenuated proteasomal activity when compared with the vector. Data are shown in Supplementary Figure S3.

### GRP78 expression in APOL1 podocytes

**Figure 5:**
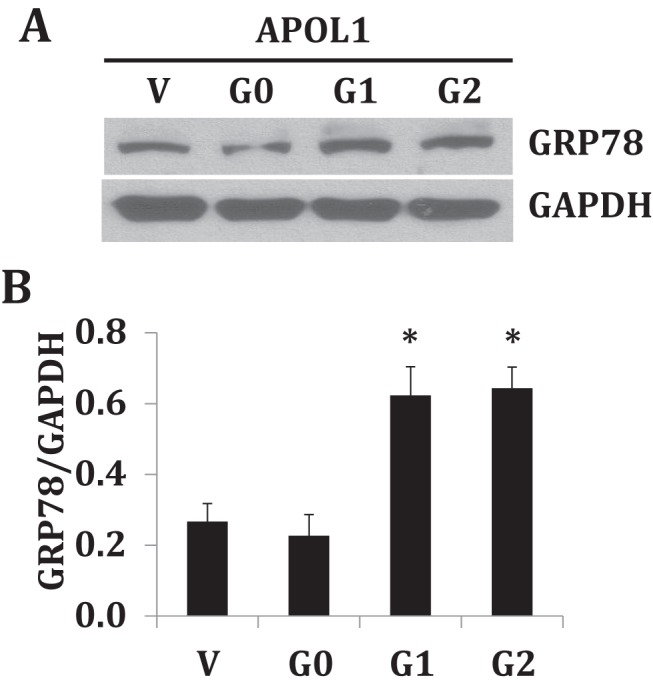
Protein GRP78 expression in APOL1 podocytes Stable podocytes were cultured and expanded at 33°C. Cells were differentiated at 37°C for 4 days. Differentiated podocytes were harvested and cell lysates were prepared in RIPA buffer. Electrophoresis of cell lysates was done by loading equal amounts of protein and transferred on to a membrane. Blots were probed with GRP78 antibody and blots were stripped and re-probed for GAPDH (**A**). Densitometric scans of Western blots (n=3) were quantified and cumulative data (mean ± S.D.) are displayed as a bar graph (B). *P<0.05 compared with G0.

There are several protein markers, which sense endoplasmic reticulum (ER) stress within a damaged cellular process. GRP78 is a chaperone protein that is up-regulated in case of ER stress and accumulation of misfolded proteins. To test GRP78 expression, cellular lysates of a vector, APOL1G0, APOL1G1 and APOL1G2 were probed with GRP78 and re-probed with GAPDH for endogeneous loading control (Figure 5A). Densitometric scans were quantified and cumulative data are shown as a bar graph (Figure 5B). Our data indicate that GRP78 is up-regulated both in APOL1G1 and APOL1G2 podocytes. However, it does not prove an accumulation of misfolded APOL1 proteins in APOL1-variant podocytes. It is possible that APOL1 risk variants lead to ER stress through the mechanism involving properly folded APOL1 proteins.

### Transcript (mRNA) expression of active misfolding markers (GRP78, XBP1, ATF6 and GADD153) in vector and APOL1 variants (G0, G1 and G2)

Total RNA was extracted from differentiated podocytes expressing vector, APOL1G0, APOL1G1 and APOL1G2. cDNAs were amplified with a specific primer for APOL1. Details of mRNA expression are shown as bar graphs in Supplementary Figure S4A–D**.**

### Bioinformatics approach to predict the effect of mutation on APOL1 stability

**Figure 6:**
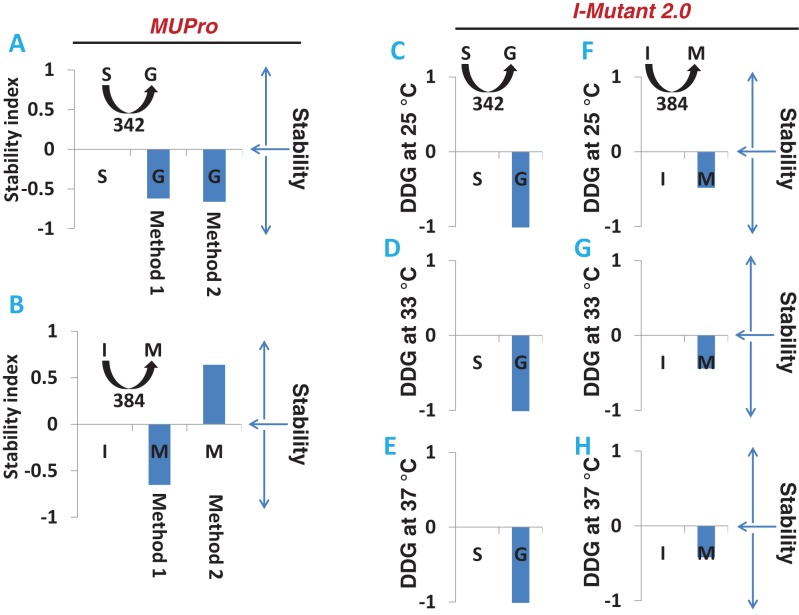
Prediction of APOL1 protein stability in APOL1G0 and APOL1G1 Amino acid sequence of APOL1G0 and APOL1G1 were analysed and compared using two different programs (MUPro and I-Mutant 2.0). MUPro compares and calculates using two methods/algorithm, APOL1G0 compared with APOL1G1 (S→G at position 342) (**A**) and APOL1G0 compared with APOL1G1 (I→M at position 384) (**B**). Amino acid sequence of APOL1G0 and APOL1G1 were analysed by another program I-Mutant2.0. Predicted outcome APOL1G0 compared with APOL1G1 (S→G at position 342) at 25°C (**C**), at 33°C (**D**) and at 37°C (**E**). The predicted outcome of APOL1G0 compared with APOL1G1 (I→M at position 384) at 25°C (**F**), at 33°C (**G**) and at 37°C (**H**).

MUPro is a program that predicts protein stability of mutated proteins. This program uses two methods: (i) support vector machines and (ii) neural networks methods. APOL1G1 has mutations at two locations (S342G and I384M). Using both methods, the S342G (Serine 342 Glycine) mutation showed predicted reduced protein stability (Figure 6A). The I384M (Isoleucine 384 Methionine) mutation compared with APOL1 G0 gave a prediction of decreased protein stability compared with APOL1 G0 by method 1 and increased protein stability by method 2 (Figure 6B).

I-Mutant2.0 is a program that yields a computed prediction of protein stability changes upon single-site mutations based on the support vector machine. This program has a prediction scale that considers different temperatures and pH milieu as well. For the position 342 S→G mutation, predicted stability scores are shown as a bar graph at 25°C (Figure 6C), at 33°C (Figure 6D) and at 37°C (Figure 6E). The results for the S342G substitution within APOL1G1 predicts reduced APOL1 protein stability compared with APOL1G0. Scores for the I384M substitution are shown as bar graphs at 25°C (Figure 6F), at 33°C (Figure 6G) and at 37°C (Figure 6H), showing reduced stability compared with APOL1G0 at all tested temperatures. Taken together, the outcomes of these prediction approaches indicate that the S342G mutations in APOL1G1 induce destabilization of APOL1 protein and the I384M also likely contributes to reduced protein stability. These results were consistent across the temperature ranges examined.

### *APOL1* mRNA transcription and stability of *APOL1* mRNA transcript within podocyte APOL1 cell lines

**Figure 7:**
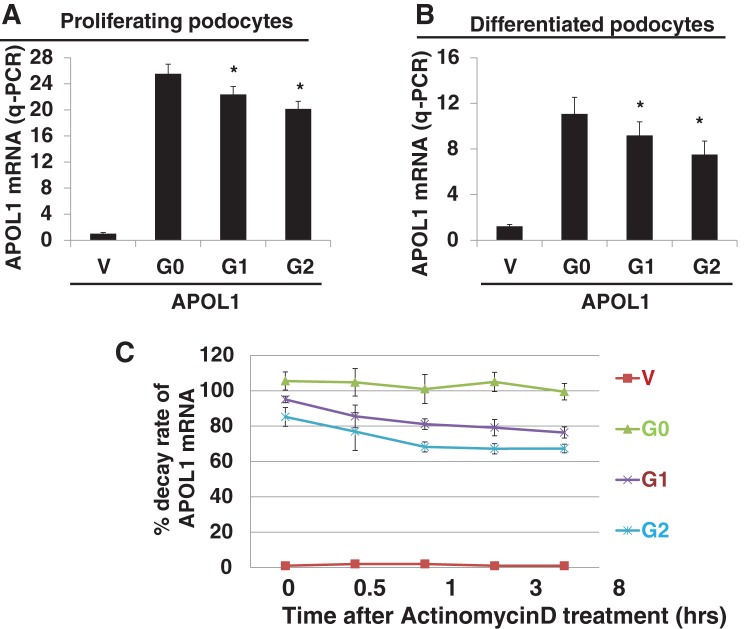
*APOL1* mRNA transcript expression and *APOL1* mRNA transcript stability within stable APOL1 podocytes Total RNA was extracted from proliferative and differentiated podocyte APOL1 cell lines using Trizol reagent. Approximately 4–5 μg RNA was used for cDNA synthesis using first-strand synthesis system (Thermo Fisher) and random hexamer primers. *APOL1* mRNA expression was evaluated by q-PCR and fold change was calculated using GAPDH as a control. Representative bar graph of proliferative cells (**A**) and differentiated cells (**B**). Data are expressed as a graph of mean ± S.D. *P<0.05 compared with G0. To evaluate stability/decay rate of *APOL1* mRNA, podocytes were pulsed with actinomycin D. Cells were harvested at time points (0, 0.5, 1, 3 and 8 h) and total RNA was extracted. RNA was converted into cDNA. q-PCR was carried out to analyse *APOL1* mRNA expression and GAPDH was used as an internal control (**C**).

DNA sequence variation may impact disturbances within to affect mRNA transcription and mRNA transcript stability. This was examined using q-PCR (real-time TaqMan) applied to cDNA reverse-transcribed from mRNA. Fold expression of *APOL1* mRNA in APOL1G0, APOL1G1 and APOL1G2 compared with vector within proliferative podocytes are represented as a bar graph (Figure 7A). Corresponding *APOL1* mRNA expression for differentiated APOL1 podocytes are demonstrated as a bar graph in Figure 7B. Taken together, these data indicate that *APOL1* mRNA transcription expression both in APOL1G1 and APOL1G2 are reduced compared with APOL1G0. We conclude that mutations in APOL1 variants result in disturbances in mRNA transcription.

**Figure 8:**
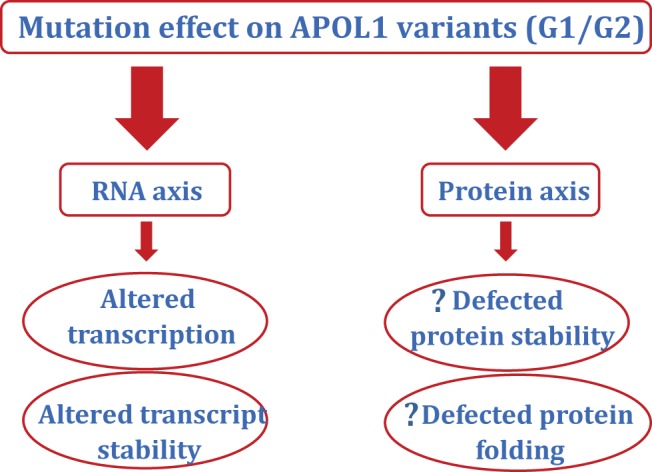
Schematic model of the effect of mutations on APOL1 variants The mutation may alter either RNA or protein pathway. Schematically, it appears that at RNA axis, APOL1 variants compromise *APOL1* mRNA transcription and APOL1 transcript stability. At protein axis, we speculate that APOL1 variants may produce defects in protein stability and protein (APOL1) misfolding.

Stability of the mRNA transcription of any gene is one of the critical factors for protein translation. Mutations can alter transcript stability. Cells transfected with vector alone, APOL1G0, APOL1G1 and APOL1G2 were pulsed with actinomycin D. mRNA was harvested at time 0, 0.5, 1, 3 and 8 h. Total RNA was extracted, reverse-transcribed to cDNA and *APOL1* mRNA expressions levels were determined by q-PCR. Cumulative data for APOL1 mRNA expression decay over time are shown in Figure 7C. Results show that *APOL1G0* mRNA destabilizes after actinomycin D pulsing over the period of time examined. Variant *APOL1G1* mRNA expression decayed 10–15% within 30 min to 3 h after actinomycin D treatment. Variant *APOL1G2* mRNA expression decayed 15–20% within 30 min to 3 h. The decay rate of *APOL1* mRNA transcript within the window of 3–8 h appears to be plateaued for both variants APOL1G1 and APOL1G2. The overall data suggest that APOL1G1 and APOL1G2 variants display compromised transcript stability most prominent in the first 0.5 h following transcription.

In brief, APOL1 variants compromise *APOL1* mRNA transcript and APOL1 transcript stability at RNA axis and defects in protein stability/misfolding

at protein axis (Figure 8).

## Discussion

The present study focusses on the impact of the mutation in APOL1 variants on deregulation of APOL1 transcription and translation. HEK 293T cells transfected with vector, APOL1G0, APOL1G1 or APOL1G2 displayed down-regulation of APOL1 expression in APOL1 variants (G1/G2) when compared with APOL1G0. An equal amount of plasmid constructs were used for transfection into 293T cells, yet APOL1 protein expression was reduced in APOL1 variants. This would suggest that mutations in APOL1 variants have an important role in protein expression and stability. These findings are consistent with other mutations altering protein expressions. Examples included mutations in GFP [5] and DRD_2_ [6]. GFP mutant is quite stable at 30°C but the same GFP mutant is unstable at 37°C, suggesting that cell culture conditions like specific temperature play an important role in protein expression and stability [8]. Stably transfected APOL1 podocytes growing at 33°C displayed decreased APOL1 expression for the APOL1 variants when compared with APOL1G0. Similarly, APOL1 podocytes cultured at 37°C displayed attenuated APOL1 expression in APOL1 variants. This suggests that the differences between APOL1G0 expression and APOL1G1 variants expression are not temperature dependent. Since APOL1 protein’s down-regulation in APOL1 variants is confirmed by three different antibody sources of APOL1 (Acris GmbH, Proteintech and Santa Cruz Biotechnology), it precludes an antibody-specific phenomenon. The proliferation and differentiation states of the podocytes did not alter this pattern of APOL1 differential expression. The APOL1 mutational variants showed consistently decreased APOL1 protein expression throughout the differentiation process. FACS analysis complemented the immunoelectrophoresis data demonstrating that APOL1 expression is decreased in cells expressing the APOL1 variants compared with APOL1G0.

Genetic mutations can induce protein misfolding and lower protein quality. Induced protein misfolding in the cellular process is sensed by unfolded protein response (UPR). The consequences of induced UPR include halting of protein translation, proteasome degradation of the misfolded protein and augmentation of molecular chaperones for protein folding. Several sensor proteins of the misfolding process are GRP78, PERK and ATF6 etc. [16,17]. In our settings, we evaluated the expression of GRP78 protein expression in APOL1 podocytes. Interestingly, we observed that expression of GRP78 is elevated in APOL1 variants compared with APOL1G0. However, these findings do not prove an accumulation of misfolded APOL1 proteins. It is quite possible that APOL1 risk isoforms lead to ER stress through mechanisms involving properly folded APOL1 proteins.

ExPASy bioinformatics programs are known to predict protein stability/instability of mutant clone compared with wild-type. We analysed and predicted protein sequence of APOL1 variant (APOL1G1) compared with APOL1G0 by the program MUPro [11], using two methods. Mutant APOL1G1 (amino acid 342, S→G) shows down-regulation of stability with both methods, predicting that this mutation causes destabilization of APOL1 protein. Mutant APOL1G1 (amino acid 384, I→M) lowers APOL1G1 protein stability as predicted by method 1, while method 2 shows augmentation in protein stabilization. We also analysed it with another program I-Mutant2.0 [12–15]. This program incorporates additional parameters to predict stability in relation to pH, temperatures, ionic strength etc. It predicts that both mutations (amino acid 342, S→G and amino acid 384, I→M) compromise protein stability prediction at all temperatures tested (25, 33 and 37°C), predicted that APOL1 protein stability is compromised with both APOL1G1 mutant substitutes compared with APOL1G0 irrespective of ambient temperature.

DNA sequence can affect the stability of transcribed mRNA. Gene mutation can lead to mRNA transcriptional instability and damaged transcription. We report that APOL1G0 mRNA expression remains consistent after pulsing with actinomycin D during the follow-up period. On the other hand, after actinomycin D pulsing, *APOL1G1* mRNA expression decayed by 10–15% within 30 min to 3 h and *APOL1G2* mRNA showed decay of 15–20% within 30 min to 3 h. Collectively, these data suggest that APOL1 mutants compromised the stability of *APOL1* mRNA transcript. Similar findings of transcript instability due to mutations have been reported in *DRD_2_* gene mutants [6]. Other than transcript stability, *APOL1* mRNA transcription level in APOL1 variants (G1 and G2) is deregulated. Interestingly, compromised *APOL1* mRNA transcription expression in APOL1 variants is also reported in stably transfected hepatocytes cell lines [10].

GRP78 is a master regulator for ER stress and UPR. It acts as a major ER chaperone with anti-apoptotic properties [18]. In the present study, both APOLG1 and APOLG2 displayed higher expression of GRP78. Thus, it appears that cells expressing APOL1-mutated proteins may sense ER stress and rise in GRP78 as a negative feedback response. At present, this is only a hypothesis and would warrant additional detailed studies.

p53 is an inducer of APOL1 [19] that has bimodal effect on autophagy; it promotes (nuclear fraction) as well as suppresses (cytoplasmic fraction) autophagy [20]. Nuclear p53 induces transcription of APOL1, which binds to phosphatic acid (a component of mTOR–phosphatic acid complex) inhibiting the mTOR activation [21]. Since mTOR is a negative regulator of autophagy, the inhibition of mTOR by APOL1–phosphatic acid complex would stimulate autophagy [22]. On the other hand, there is enough evidence suggesting the repressive role of cytoplasmic p53 on autophagy in several cancer cell lines. For example, inhibition of p53-promoted autophagy in enucleated cells but cytoplasmic p53 could repress the enhanced autophagy in *p53^−^^/^^−^* cells [20]. Similarly, starvation and rapamycin-stimulated proteasomal-mediated degradation of p53 but stimulated autophagy; additionally, inhibition of p53 degradation prevented the activation of autophagy in several cell types [20]. APOL1G0 has also been reported to induce autophagic cell death in several cell types [22]. Since deletion of the BH3 only lipid binding protein fraction of APOL1 provided protection against APOL1 induced autophagic cell death, this fraction of APOL1 has been implicated for this property [19].

Mutations in APOL1 may lead to gain or loss of function. In earlier studies, we and others have shown a gain of activity in cell injury models with a greater effect of G1 and G2 compared with G0 (Lan et al. 2014). These findings are consistent with the observation that the APOL1 gene product is dispensable for human health, except under trypanosomal or other pathogen infection, and in fact is absent from most species other than humans and some higher primates [23]. Therefore, the finding of diminished levels of APOL1 protein expression and mRNA stability for APOL1 G1 and G2 compared with G0 is not a plausible explanation for the pathogens of cell injury or for kidney disease risk. Rather, these findings reflect the functionality of the mutations and may be an early onset response to cell injury. A potential limitation of the current study is that APOL1 is toxic to cells. However, the lower levels of mRNA and proteins are not likely attributed to overall cell loss or injury, since measurements were appropriately normalized in each case. If it is a gain of function, enhanced autophagy would lead to enhanced degradation of mutated proteins leading to attenuated protein expression of APOL1 variants. It would also be a negative feedback attempt by podocytes to decrease the expression of proteins, which are toxic to cells. It will be important to study this aspect of mutated APOL1G1 and APOL1G2 in future studies.

We conclude that mutations in APOL1 variants lead to damaged and unstable APOL1 protein expression. Whether protein misfolding and/or autophagy are contributing to this modulation would warrant further studies.

## Abbreviations

APOL1: apolipoprotein L1
BH3: Bcl-2 homology domain 3
DRD_2_: dopamine receptor D_2_
ER: endoplasmic reticulum
EV: empty vector
HEK: human embryonic kidney
PERK: protein kinase RNA-like endoplasmic reticulum kinase
PGP-3: p-glycoprotein-3
UPL: universal probe library
UPR: unfolded protein response
XBP1: x-box binding protein 1
